# Immunopathological Inflammation in the Evolution of Mucositis and Peri-Implantitis

**DOI:** 10.3390/ijms232415797

**Published:** 2022-12-13

**Authors:** Varvara Labis, Ernest Bazikyan, Svetlana Sizova, Vladimir Oleinikov, Andrey Trulioff, Maria Serebriakova, Igor Kudryavtsev, Olga Zhigalina, Dmitry Khmelenin, Irina Dyachkova, Denis Zolotov, Victor Asadchikov, Alexey Volkov, Sergey Khaidukov, Ivan Kozlov

**Affiliations:** 1 Stomatology Faculty, A.I. Yevdokimov Moscow State University of Medicine and Dentistry, 20, p. 1 Delegatskaya St., 127473 Moscow, Russia; 2 Department of Biomaterials and Bionanotechnology, Shemyakin & Ovchinnikov Institute of Bioorganic Chemistry RAS, 16/10 Miklukho-Maklaya St., 117997 Moscow, Russia; 3 Federal Research and Clinical Center of Physical-Chemical Medicine of Federal Medical Biological Agency, 1A Malaya Pirogovskaya St., 119435 Moscow, Russia; 4 Federal State Budgetary Scientific Institution “Institute of Experimental Medicine”, 12, Acad. Pavlov St., 197022 Saint-Petersburg, Russia; 5 School of Biomedicine, Far Eastern Federal University, 10 Ajax Bay, Russky Island, 690922 Vladivostok, Russia; 6 Federal Scientific Research Centre “Crystallography and Photonics” Russian Academy of Sciences, 59 Leninskiy Prospekt, 119333 Moscow, Russia; 7 Department of Machine-Building Technologies, Bauman Moscow State Technical University, 5/1 2-ya Baumanskaya St., 105005 Moscow, Russia; 8 Federal State Budgetary Institution “National Medical Research Center for Traumatology and Orthopedics Named after N.N. Priorov” of the Ministry of Health of the Russian Federation, 10, Priorova St., 127299 Moscow, Russia; 9 Institute of Professional Education, I.M. Sechenov First Moscow State Medical University, 8-2 Trubetskaya St., 119991 Moscow, Russia

**Keywords:** nanoparticles, immunopathological inflammation, cell activation, early and late cell apoptosis, THP-1 cell line, X-ray microtomography (XMCT), X-ray fluorescence analysis (XRF), electron microscopy, dynamic light scattering (DLS), flow cytometry (FC)

## Abstract

The purpose of this study was to provide an immuno-mediated substantiation of the etiopathogenesis of mucositis and peri-implantitis based on the results of experimental, laboratory and clinical studies. The biopsy material was studied to identify impregnated nanoscale and microscale particles in the structure of pathological tissues by using X-ray microtomography and X-ray fluorescence analyses. Electron microscopy with energy-dispersive analysis identified the composition of supernatants containing nanoscale metal particles obtained from the surfaces of dental implants. The parameters of the nanoscale particles were determined by dynamic light scattering. Flow cytometry was used to study the effect of nanoscale particles on the ability to induce the activation and apoptosis of immunocompetent cells depending on the particles’ concentrations during cultivation with the monocytic cell line THP-1 with the addition of inductors. An analysis of the laboratory results suggested the presence of dose-dependent activation, as well as early and late apoptosis of the immunocompetent cells. Activation and early and late apoptosis of a monocytic cell line when THP-1 was co-cultured with nanoscale metal particles in supernatants were shown for the first time. When human venous blood plasma was added, both activation and early and late apoptosis had a dose-dependent effect and differed from those of the control groups.

## 1. Introduction

It is well known that mucositis and peri-implantitis are complications of dental implantations that are associated with the development of a chronic inflammatory destructive process in the soft and bony tissues of the jaw. Initially, the causes of their occurrence were considered from the perspective of an interaction between the human body and a single certified medical device with a patented surface treatment, without taking the possibility of the emission of particles of different sizes into account.

It is known from the literature that the cause of mucositis and peri-implantitis can include titanium ions and nanoparticles as degradation products of medical products under conditions of functional load [1,2,3,4,5,6,7,8,9,10]. In 2021, works appeared that were devoted to the genetic and immunological aspects of the interaction of titanium nanoparticles with macrophages and studies of gene expression during their co-culture [11].

Previously, the authors of this article suggested that nanoscale metal particles (NSMP) in high concentrations were the main trigger for chronic immunopathological inflammation in the bone bed and oral mucosa tissues. Their accumulation in tissues up to the critical mass called the critical dose of nanoscale metal particles (CDNanoMP) leads to the early death of cells of the innate immune system, which is obviously the main reason for the coagulation of the accumulated nanoparticles with the formation of micron-sized complexes against the background of chronic immunopathological inflammation. The current immunological concept involves the osseointegration and disintegration of dental implants. In our earlier studies [12,13,14,15,16], the process of osseointegration of dental implants was the result of the recognition of complexes of nanoscale particles covered by the protein capsid shell of blood plasma with an altered tertiary structure, in which serum proteins were the main component.

During the second orthopedic stage in the classical technique of dental implantation, the appearance of a functional load facilitates the release of nanoparticles and the formation of microparticles [17]. Presumably, over time, there is a disruption of the immune system’s tolerance and activation of antibody synthesis caused by the accumulation of a critical mass of particles, the early death of immunocompetent cells and the onset of endogenous infection. The involvement of oral microbiota as a secondary link in the initiation of a chronic inflammatory process (i.e., mucositis) aggravates the course, translating it into a chronic immunopathological disease, namely peri-implantitis.

The aim of these multidisciplinary studies was to investigate the influence of nano- and microscale particles on the formation of the conditions for the pathological inflammatory process in tissues. For this purpose, the following methods were used: X-ray microtomography, X-ray fluorescence analysis, electron microscopy, dynamic light scattering and flow cytometry. The possibility of the activation and apoptosis of immunocompetent cells, such as monocytes, was studied in an experimental model. Co-culture of human monocytic leukemia cells (the THP-1 cell line) with NSMP obtained from the surfaces of dental implants made it possible to assess the viability of immunocompetent cells. The use of flow cytometry made it possible to assess the probability of an interaction between immunocompetent cells and the titanium alloy, which was previously considered to be “bioinert”, and to identify the effects of the interaction with certified medical products.

## 2. Results and Discussion

### 2.1. Results of Soft Tissue Biopsy Specimens

#### 2.1.1. Results of Histological Studies

**Figure 1:**
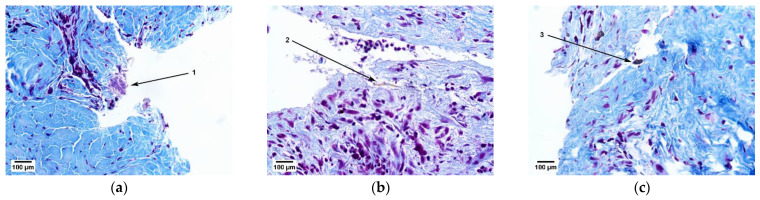
Results of the histological study of soft tissue samples of the patient’s fibrous capsules (Mallory staining): (**a**) granulation tissue at different stages of maturation; (**b**) maturing dense fibrous connective tissue; (**c**) foreign particles.

A histological study of the soft tissue samples of the patient’s fibrous capsules above the cervical area of the Nobel Replace (Danaher Corporation, Washington, DC, USA) dental implants revealed bacterial colonies (Figure 1a, Item 1), pieces of food (Figure 1b, Item 2) and foreign inclusions in the form of irregular black spots (Figure 1c, Item 3), which required clarification of their origin by X-ray microtomography and elemental analysis.

#### 2.1.2. Results of X-ray Studies

**Figure 2:**
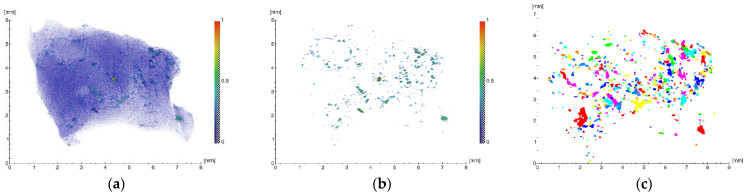
Three-dimensional reconstruction of the specimen (fibrous capsule): (**a**) initial image with foreign inclusions; (**b**) image of the foreign inclusions after the segmentation procedure; (**c**) the resulting projection of the foreign inclusions after the segmentation procedure.

The results of the tomographic studies are shown in Figure 2.

To quantify the microinclusions contained in the sample, the obtained 3D image was processed, which included threshold filtering, binarization and labeling. The resulting projection of all the inclusions after this approach is shown in Figure 2c.

During the analysis, the following parameters were determined: spatial distribution, dimensions (equivalent diameter and volume) and the average linear absorption coefficients (Table 1) of the foreign inclusions detected in the biological specimen. The equivalent diameters of the inclusions (*EqD*), representing the diameters of a sphere of the same volume, were calculated according to the following equation:

(1) $$EqD=\sqrt[{3}]{\frac{6\times \mathrm{Volume}}{\pi }}$$

It should be noted that a large number of microinclusions with sizes from ~11 to 235 μm with linear absorption coefficients µ from ~0.22 to 0.6 mm^−1^ were detected (Table 1). Thus, the concentration of foreign microscale particles (MSMP) in the analyzed volume of the fibrous capsule was estimated according to the equation:

(2) $$C_{B}=\frac{N_{B}}{V}$$

where *N_B_* is the number of particles and *V* is the volume. For the volume of the studied sample (~3.3 × 10^10^ µm^3^) and the number of detected microparticles (~700), the concentration was ~4.6 × 10^7^ µm^−3^.

**Figure 3:**
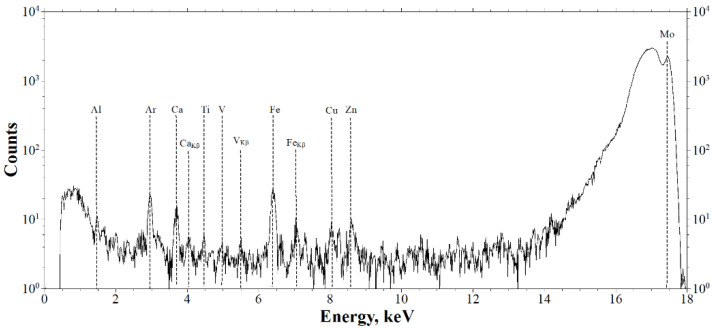
The XRF spectra of the elemental composition of the sample (the fibrous capsule adjacent to the Nobel Replace system implant).

The results of the study of the elemental composition inside the soft tissue biopsy specimens (clinical case) by XRF are presented in Figure 3. The interpretation of the spectra revealed the presence of the following elements: Al, Ca, Ti, V, Fe, Cu and Zn. The peaks of Ar (air) and Mo (the anode of the X-ray tube) were from the instrument.

In the earlier studies [18] of the surfaces of dental implants of two systems (Nobel Replace (Danaher Corporation, Washington, DC, USA) and Alpha-Bio (Alpha-Bio Tec Ltd., Petach Tikva, Israel)), as well as NSMP and MSMPs found in the soft tissues surrounding the dental implants, it was found that the microparticles of the Nobel Replace implants were mainly titanium dioxide of both modifications, namely rutile (TiO_2_, space group P4_2_/mnm) and anatase (TiO_2_, space group I4/amd). In the case of the Alpha-Bio implants, in addition to titanium dioxide and titanium nitride, aluminum oxides were present. The elemental composition of the nanoscale particles showed the presence of elements such as Fe, V, Ca, Na, Cl, S, Si and P. There was a correlation between the established composition of the metal particles and the composition declared by the manufacturers of these dental systems [19,20]. The presence of metal trace elements in the composition of the soft tissues surrounding the dental implants can only be a correlation of their introduction and mechanical loading on the osseointegrated implants.

In addition, it was previously discovered [18] that the surface morphology of the implants of both systems differed significantly. The nature of the relief of the Alpha-Bio dental implants was such that NSMP emissions from its surface into the adjacent tissues were more likely than from the surface of the Nobel Replace implant.

In the process of the clinical case studies in the present work, XMCT and XRF were used to identify the impregnated MSMP in the structure of the pathological areas of tissue (Figure 2 and Figure 3). It was also found that the elements revealed in the sample, namely Al, Ti and V, corresponded to the composition of the titanium compound used in the production of the Nobel Replace dental implant systems (Figure 3, [19,20]).

### 2.2. Results of the Supernatant Studies

A comparison of the results of clinical studies of soft tissue biopsy specimens with analyses of the composition of the supernatant obtained from the surface of newly certified medical devices from the same manufacturer made it possible to assess the involvement of NSMP in the development of chronic inflammatory complications. Dental implants from another leading manufacturer were used for comparison and to obtain reliable statistical data.

#### 2.2.1. STEM and EDX Results of the Supernatants Obtained from the Surfaces of the Nobel Replace and Alpha-Bio Dental Implant Systems at a Dilution Ratio of 1.0 before Filtration

**Figure 4:**
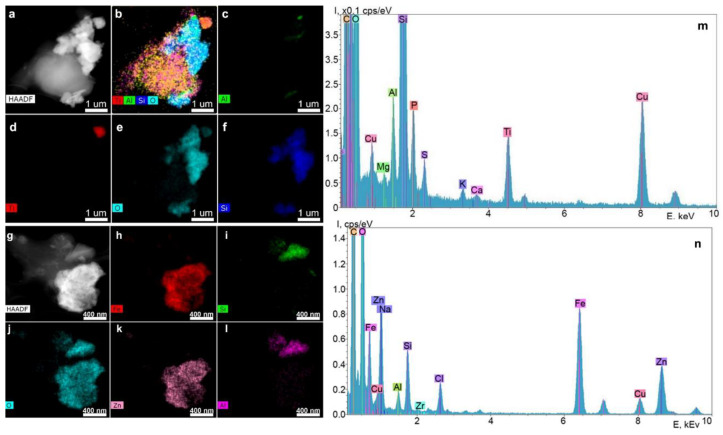
STEM image of particles with z-contrast (**a,g**), chemical element distribution maps (**b**–**f**,**h**–**l**) and the corresponding energy-dispersive spectrum (**m,n**).

Figure 4 shows the results of the analysis of particles contained in the supernatants obtained from the surface of Nobel Replace dental implants using STEM and EDX.

According to the chemical element distribution maps, the sample contained particles of Fe, Zn, Al, Zr, Si oxides and Ti. The particle sizes ranged from a few tens of nanometers to a few micrometers, and the shape was random. Elements such as P, Mg, S and K were distributed uniformly over the particle area shown in the STEM images. The Cu peaks for all spectra obtained by transmission electron microscopy appeared to be caused by the presence of the copper grid on which the particles under study were placed. Since the particles were on a carbon substrate, it was not possible to determine the carbon content of the particles.

**Figure 5:**
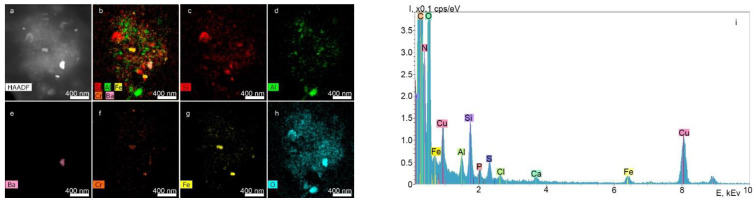
STEM image of particles with z-contrast (**a**), chemical element distribution maps (**b**–**h**) and the corresponding energy-dispersive spectrum (**i**).

Figure 5 shows the results of the analysis of the particles contained in the supernatants obtained from the surfaces of the Alpha-Bio dental implant system by STEM and EDX.

As in the previous case, Ti, Al, Fe and Si oxides up to several micrometers in size were revealed most often. There were also single particles of Ba, Cr, Zn, Mg, Cl and N, which were distributed uniformly over the image’s area.

#### 2.2.2. Results of DLS of Supernatants Obtained from the Surfaces of the Nobel Replace and Alpha-Bio Dental Implant Systems at a Dilution Ratio of 1.0 after Filtration

The parameters of NSMP in the supernatants of the Nobel Replace and Alpha-Bio systems, determined by DLS at a dilution ratio of 1.0, were as follows:

- Frequency of occurrence: Nobel Replace, 6.4 kcps; Alpha-Bio, 6.0 kcps;
- NSMP size: Nobel Replace, 103.31 nm; Alpha-Bio, 187.75 nm;
- Polydispersity: Nobel Replace, 0.744%; Alpha-Bio, 0.480%.

#### 2.2.3. Results of Co-Culturing Supernatants Obtained from the Surfaces of Nobel Replace and Alpha-Bio Dental Implants with Lipopolysaccharide Induction and the Addition of Fresh Human Blood Plasma

**Figure 6:**
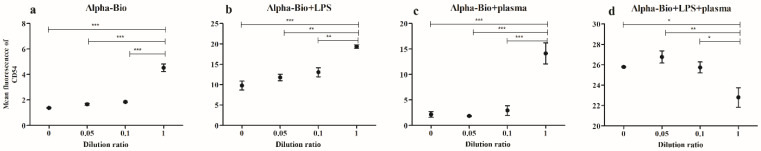
Changes in the expression level of CD54 by THR-1 cells after 24 h of incubation with NSMP from the Alpha-Bio system: (**a**) incubation with NSMP; (**b**) incubation with NSMP and LPS; (**c**) incubation with NSMP and blood plasma from healthy donors; (**d**) incubation with NSMP, LPS and blood plasma from healthy donors. Statistical significance levels: * *p* < 0.05, ** *p* < 0.01, *** *p* < 0.001.

**Figure 7:**
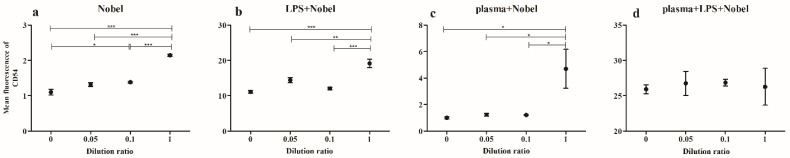
Changes in the expression level of CD54 molecules by THP-1 cells after 24 h of incubation with NSMP from the Nobel Replace system: (**a**) incubation with NSMP; (**b**) incubation with NSMP and LPS; (**c**) incubation with NSMP and blood plasma from healthy donors; (**d**) incubation with NSMP, LPS and blood plasma from healthy donors. Statistical significance levels: * *p* < 0.05, ** *p* < 0.01, *** *p* < 0.001.

The activation of THP-1 line cells under the influence of lipopolysaccharide (LPS) on the two dental implant systems was estimated according to the expression level of CD54 on the cells’ surface (Table 2 and Table 3, Figure 6 and Figure 7).

The data presented in Table 2 and Figure 6a show that incubation with the NSMP of the Alpha-Bio system led to a significant increase in the expression of CD54 on the surface of the THP-1 cells, and there was a tendency for this process to show a dose-dependent nature. A similar effect was observed when the cells were co-incubated with NSMP and LPS or human blood plasma (Figure 6b,c). It should be noted that the incubation of THP-1 with LPS caused a significant increase in CD54 expression compared with the control group, in which a solution of phosphate buffered saline (PBS) was added, irrespective of the concentration of NSMP (p<0.001).

Stimulation of the cells simultaneously with LPS and human blood plasma also caused a significant increase in the expression of CD54 (p<0.001). Injection of low NSMP concentrations led to an additional insignificant variation in the number of molecules on the cells’ surface, while the maximum concentration significantly reduced the level of cell activation (p<0.001) (Figure 6d). The greater contribution to the total variance was from the presence of a co-stimulator (PBS, LPS or human blood plasma), namely 86.76% (F3.32=646.22, p<0.0001), and 4.94% of the variance was provided by the change in the concentration of nanoparticles (F3.32=36.83, p<0.0001).

The data obtained after incubation of the cells with NSMP of Nobel Replace are shown in Table 3 and Figure 7. In the data presented here, one can note a trend similar to that mentioned above. When the cell culture was incubated with NSMP only, reliable differences were observed only when the maximum concentration was applied (Figure 7a). Against the background of LPS stimulation, the level of expression significantly changed, starting from the lowest NSMP concentration (Figure 7b), while human plasma completely dampened their effect (Figure 7c,d). Assessment by dispersive analysis showed that the nanoparticles contributed only 1.4% (F3.32=8.93, p=0.0002) to the total variance, whereas the addition of co-stimulants contributed 95.49% (F3.32=607.40, p<0.0001).

**Figure 8:**
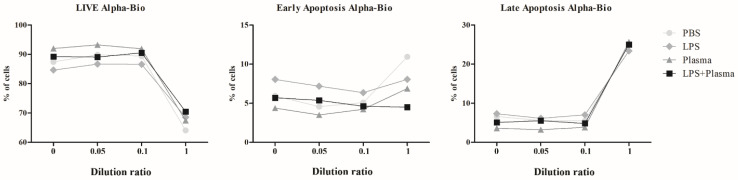
Viability of THP-1 cells after 24 h of incubation with NSMP from the Alpha-Bio system. PBS, incubation with NSMP and PBS as a co-stimulator; LPS, incubation with NSMP and PBS as a co-stimulator; Plasma, incubation with NSMP and blood plasma from healthy donors as a co-stimulator; LPS + Plasma, incubation with NSMP and a combination of LPS and blood plasma from healthy donors as co-stimulators.

**Figure 9:**
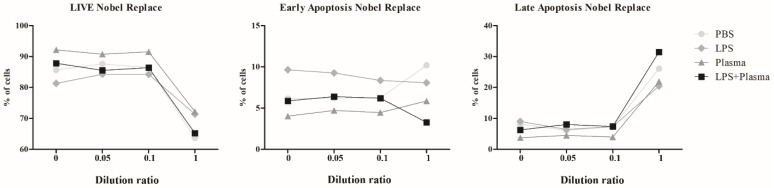
Viability of THP-1 cells after 24 h of incubation with NSMP from the Nobel Replace system. PBS, incubation with NSMP and PBS as a co-stimulator; LPS, incubation with NSMP and PBS as a co-stimulator; Plasma, incubation with NSMP and blood plasma from healthy donors as a co-stimulator; LPS + Plasma, incubation with NSMP and a combination of LPS and blood plasma from healthy donors as co-stimulators.

The results of estimating the cell viability of the THP-1 line under the influence of NSMP from the two dental implant systems in this study are shown in Table 4 and Table 5, Figure 8, Figure 9, Figures S1 and S2.

During the co-culture of the THP-1 cells with NSMP obtained from the surface of Alpha-Bio dental implants, a statistically significant decrease in cell viability against the background of exposure to high concentrations of the particles was observed, which did not depend on the presence of co-stimulants. The maximum contribution (90.02%) to the total dispersion was made by the particle concentration factor (F3.32=220.21, p<0.0001), while the co-stimulation factor had a much smaller, although significant, impact on the total dispersion, namely 3.22% (F3.32=7.87, p=0.0005). The effect of the interaction between particles and co-stimulation on cell viability did not contribute significantly to the overall dispersion (F9.32=1.96, p=0.0786).

Cell death processes at early stages (early apoptosis) were determined by both the NSMP concentration factor (contribution to total dispersion = 26.32% (F3.32=22.31, p<0.0001)) and the co-stimulation factor (31.06% (F3.32=26.33, p<0.0001)), as well as their interaction (30.05% (F9.32=8.49, p<0.0001)). Meanwhile, the stage of death (late apoptosis and necrosis), implying cell membrane destruction, was significantly affected only by the concentration of nanoparticles from the Alpha-Bio implant system (92.78% (F3.32=181.09, p<0.0001)).

In the case of co-culturing THP-1 cells with NSMP obtained from the surface of the Nobel Replace dental implant system, a statistically significant decrease in cell viability against the background of exposure to high concentrations of the particles was also observed. The greatest contribution to the total variance in the estimation of the proportion of living cells was made by the particle concentration factor (80.02%) (F3.32=117.88, p<0.0001). A lower, but also significant, influence on viability was seen for the co-stimulation factor (7.89% (F3.32=11.62, p<0.0001)) and the interaction of both of these factors (4.85% (F9.32=2.383, p=0.0342)).

NSMP from the Nobel Replace system did not make a significant contribution to the change in the proportion of cells in early apoptosis (0.89% (F3.32=0.50, p=0.6875)); however, they were a determining factor for the late stages of cell death (84.42% (F3.32=129.42, p<0.0001)). For early apoptosis, additional stimulation was more of a determinant factor (contribution to total variance = 52.41% (F3.32=29.26, p<0.0001)), and the combined effect of co-stimulation and nanoparticles was 27.59% (F9.32=5.14, p=0.0003). Co-stimulation contributed 4.01% to the total variance of the results obtained through the evaluation of late apoptosis and necrosis (F3.32=6.15, p=0.0020), and the interaction of nanoparticles and stimulants contributed 4.61% (F3.32=2.36, p=0.0361).

**Figure 10:**
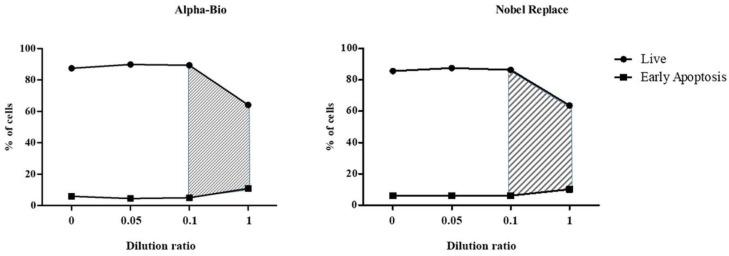
Range of the critical dilution ratios of supernatants containing NSMP from Nobel Replace and Alpha-Bio for the development of apoptosis.

The FC method showed that NSMP led to the activation of immunocompetent cells, as well as early and late apoptosis up to necrosis. It was shown that the double stimulation of immunocompetent cells by both nanoparticles and lipopolysaccharide enhanced cell death. The range of critical supernatant dilution ratios for the two dental implant systems was identified (Figure 10). A change in the dilution factor from 0.1 to 1, which was associated with an increase in the incidence of NSMP, led to early apoptosis and necrosis in immunocompetent cells.

Stepwise multidisciplinary research seems to be the most promising approach to studying and substantiating the pathogenesis of peri-implantitis from the perspective of a local chronic immunopathological inflammatory process.

## 3. Materials and Methods

### 3.1. Clinical Case

**Figure 11:**
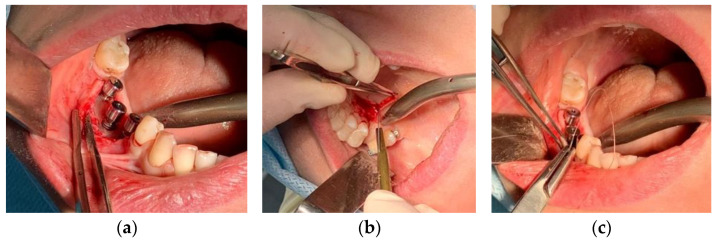
Stages of dental implant surgery according to the classical two-stage method using the Nobel Replace implant system: (**a**) condition of the bone bed in the area of the osseointegrated dental implants at the time of the second surgical stage of dental implantation; (**b**) extraction of the connective tissue graft from the area of the mucous membrane of the alveolar process of the maxilla in the projection of the hard palate; (**c**) soft tissue surgery using a connective tissue graft taken from the region of the hard palate.

Patient S. (female) was 47 years old. At the Department of Oral Surgery No. 2 of the Moscow State University of Medicine and Dentistry, classical two-stage dental implant surgery was performed using the Nobel Replace implant system for the patient S. After three months, the second surgical stage of dental implantation requiring the installation of gum shapers was performed. During this stage, fibrous capsules above the surface of the right dental implants in Projections 45, 46 and 47, as well as bone resorption in the cervical area of dental implants were revealed at a depth of 3 mm (Figure 11a). It should be noted that the patient did not use a temporary removable prosthesis, and the wound’s surface, after dental implantation, healed with primary tension under the protection of complex antibacterial and anti-inflammatory therapy without complications. Soft tissue biopsy specimens, consisting of fibrous capsules adjacent to the cervical zone of the Nobel Replace dental implant system, were submerged in 10% formalin. Both the microtomographic X-ray images and the elemental composition of the external inclusions were then studied. Bone tissue defects were closed with connective tissue grafts (Figure 11b,c).

### 3.2. Histological Studies

Histological studies of the soft tissue samples (fibrous capsules) located above the cervical area of the dental implants were performed after the second surgical stage of dental implantation and biopsy sampling. The material was placed in a 10% solution of neutral formalin for 72 h, after which, the tissue samples were washed in running water for 2 h. After standard histological preparation, the tissue samples were embedded in paraffin (Histomix, Biovitrum, St. Petersburg, Russia), using histological pouring rings (Biovitrum, St. Petersburg, Russia). Serial and semi-serial slices were made from the blocks on a Microm microtome (3–7 μm). The specimens were stained by Mallory technique (Biovitrum, St. Petersburg, Russia) to reveal specific processes of connective tissue formation.

### 3.3. X-ray Microtomography (XMCT)

X-ray absorption microtomography was used to identify external inclusions in the specimens under study, which were a part of the fibrous capsule adjacent to the cervical zone of the Nobel Replace dental implant system, and to determine their spatial distribution and size. The measurements were performed on a “TOMAS” X-ray microtomograph using an X-ray tube with a molybdenum anode (energy, 17.5 keV) and a pyrolytic graphite monochromator single crystal (the size of the beam on the object was about 1 cm). The parameters of the experiments were as follows: source–sample distance, 1.2 m, sample–detector distance, 0.02 m. The probing conditions were an accelerating voltage of 40 kV and a current of 40 mA. The sample was rotated around a fixed vertical axis and 400 projections were measured with a step of 0.5 degrees and 4 s of exposure time per frame. A high-resolution X-ray detector, XIMEA-xiRay11 (XIMEA, Marianka, Slovakia), with a 9 μm pixel size and a 36 × 24 mm field of view was used for the measurements. The reconstruction was performed by the algebraic method using ASTRA Toolbox software v. 2.1 [21].

### 3.4. X-ray Fluorescence Analysis (XRF)

The elemental composition of the sample (part of the fibrous capsule adjacent to the Nobel Replace dental implant system) was determined by X-ray fluorescent analysis on an X-ray microtomograph using an X-123SDD detector-spectrometer (Amptek, Bedford, MA, USA). The radiation source was an X-ray tube with a molybdenum anode, of which the radiation was monochromatized by a single reflection from a highly perfect symmetric silicon single crystal, Si(111). The radiation energy of 17.47 keV corresponded to the characteristic K_α1_ line of molybdenum. The lower threshold of the measurements was limited by the parameters of the detector’s sensitive element of ~1 keV, and the energy resolution was ~150 eV. The parameters of the experiment were as follows: a source–sample distance of about 1 m and a sample–detector distance of about 0.02 m. The size of the probing beam on the sample was 1 × 3 mm (h × v). The probing conditions were an accelerating voltage of 40 kV and a current of 40 mA. The exposure time was 600 s.

### 3.5. Electron Microscopy

The surface composition of the dental implant was studied by transmission electron microscopy (TEM), scanning transmission electron microscopy (STEM) with z-contrast and energy-dispersive X-ray (EDX) analysis using a transmission electron microscope with field emission (ThermoFisher Scientific, Osiris, Waltham, MA, USA) at an accelerating voltage of 200 kV. The transmission electron microscope was equipped with a special system of detectors allowing us to obtain maps of the chemical elements’ distribution across a large area in a few minutes.

For the TEM analysis, supernatant samples containing nanoscale oxide film elements obtained from the surface of the dental implants were prepared by the method described in the patent [22]. Supernatants with nanoscale particles obtained from the surface of the two dental implant systems (Nobel Replace and Alpha-Bio) were deposited on copper grids with a thin substrate of amorphous carbons.

### 3.6. Experimental Laboratory Study

#### 3.6.1. Preparation of a Suspension of Nanoscale Particles from the Surface of the Nobel Replace and Alpha-Bio Dental Implant Systems

To obtain supernatants containing NSMP used in the experimental part of the study, 40 units of the Nobel Replace and Alpha-Bio dental implant systems were incubated in double-distilled water for 5 days in a CO_2_ incubator. Nanoscale particles were obtained by the method described in the patent [22]. After incubation, tubes containing the implants were treated with ultrasound at a frequency of 35 kHz for 20 min. Under laminar conditions, the implants were removed from the tubes, the water was evaporated, and the remaining sediment containing the nanoscale particles was diluted in a solution of phosphate buffered saline (PBS), adjusting the concentration to 1/20 of the original volume. The resulting supernatant samples were filtered through a Millipore syringe nanofilter (maximum pore diameter D = 0.22 μm).

#### 3.6.2. Dynamic Light Scattering (DLS)

The method of dynamic light scattering (photon correlation or quasi-elastic light scattering) can be used to measure objects from 1 nm to 10 µm in size, which allowed us to detect the yield of NSMP and MSMP (microscale metal particles) from the objects of this study [17].

To identify nanoscale particles from the surfaces of the two dental implant systems in the supernatants [23] and to determine their size, a study was performed on a 90 Plus Particle Size Analyzer (Brookhaven Instruments Corporation, Holtsville, NY, USA) in multimodal mode using the automatic 90Plus/BI-MAS and “dust cut-off” functions that allow the user to subtract very large objects, particularly dust. The filter’s value was 20. The measurements were recorded at a temperature of 25 °C and a 661 nm laser with a fixed light scattering angle.

Using the DLS method, three parameters were measured: the NSMPs’ diameter (D, nm), the frequency of the NSMPs’ occurrence in the supernatants (ACR, kcps) and the index of heterogeneity of the NSMP’s distribution in the supernatants, known as NSMP polydispersity (PD, %). The MS Excel software package (Microsoft Office 2016) was used to process the statistical data for the two systems, i.e., Nobel Replace and Alpha-Bio.

#### 3.6.3. Cell Cultivation

The THP-1 suspension cell line (human monocytic leukemia cells) was chosen as the object of the present study. The THP-1 cells were cultured in RPMI-1640 medium (Biolot, St. Petersburg, Russia) in the presence of 10% inactivated fetal calf serum (Biolot, St. Petersburg, Russia), 50 µg/mL gentamicin (Biolot, St. Petersburg, Russia) and 2 mM L-glutamine (Biolot, St. Petersburg, Russia). Reseeding was performed every 2–3 days. The cells were incubated at 37 °C in a 5% CO_2_ atmosphere. Plastic vials with a volume of 50 mL (Sarstedt, Numbrecht, Germany) were used. For the experiments, 250 µL of the cell suspension (1 × 10^6^ cells per mL) in RPMI-1640 culture medium was added to 96-well flat-bottom plates (Sarstedt, Numbrecht, Germany). Supernatants of the NSMP of the two dental implant systems at three different dilution ratios (0.05, 0.1 and 1.0 of the initial suspension’s concentration) were added to the wells to stimulate the cells. PBS was used as a negative control. In addition, supernatants containing NSMP were added to cells against the background of adding lipopolysaccharide and blood plasma from healthy donors separately and in combination. The incubation time in the presence of the stimulants was 24 h at 37 °C in a 5% CO_2_ atmosphere. After the incubation, the cells in each well were divided into two parts; the first part was used to estimate the viability and the second one was used to estimate the level of cell activation in the sample.

#### 3.6.4. Assessment of Cell Viability after Interactions with NSMP

Cell viability was assessed using the DNA-binding dyes YO-PRO-1 (iodide) and PI (Propidium Iodide Solution) [24]. A specific feature of these substances is their ability to bind to nucleic acids, which is accompanied by an accumulation of dyes in the cell and, as a result, an increase in the fluorescence level. A significant difference between these dyes is the way they penetrate through the bilipid layer of the cell membrane, which is not penetrable by them. P2RX7 ligand-dependent ion channels belonging to the purinoreceptor family are responsible for the accumulation of YO-PRO-1 inside the cytoplasm [25,26]. In living cells, these channels are not active or have a weak ability to transport the dye through the membrane, and YO-PRO-1 is not accumulated. When apoptosis is triggered, their activation coincides with the disturbance of the asymmetry of the lipid composition of the surface membrane. This makes it possible to consider the accumulation of YO-PRO-1 in cells as an event that is characteristic of the “early” stages of apoptosis. PI does not have any membrane-integrated specific transporters and can penetrate into the cytoplasm and nucleus only through damaged/fragmented membranes, which usually takes place in the final stages of apoptosis during the formation of apoptotic cells or during cell necrosis. Thus, the live cells in the sample will not be stained with either of the above-mentioned dyes. Cells that have entered apoptosis will only be positive for YO-PRO-1, and those in the later stages of apoptosis will be effectively stained with both dyes.

Cell staining was performed in 12 × 75 mm cytometric test tubes (Beckman Coulter, Brea, CA, USA). For this purpose, solutions of YO-PRO-1 (Invitrogen, Waltham, MA, USA) and PI (Sigma-Aldrich, Burlington, MA, USA) were added to 100 µL of the cell suspension (5 × 10^5^ cells/mL), obtaining a final concentration of 250 nM and 1 μg/mL, respectively. The staining was performed at room temperature for 5 min in a light-protected place.

#### 3.6.5. Assessment of Cell Activation after Interactions with NSMP

The level of cell activation was estimated by using monoclonal antibodies against the aberrant CD54 marker conjugated with isothiocyanate fluorescein FITC (Cat. No. IM0726U, manufactured by Beckman Coulter, Brea, CA, USA). A two-component conjugate of mouse IgG1 immunoglobulin with FITC and phycoerythrin PE (Cat. No. A07794, manufactured by Beckman Coulter, Brea, CA, USA) was used as an isotypic control.

Antibody staining was performed according to the manufacturer’s recommendations. The incubation time for the cells with the antibodies was 15 min at room temperature in a light-protected place. At the end of incubation, the samples were washed once with excess PBS at 330 g for 7 min, after which the supernatant was removed and the cell sediment was resuspended in PBS at pH 7.2–7.4, and a DAPI fluorescent dye solution (Sigma, Saint Louis, MO, USA) was added to the samples at a final concentration of 1 µg/mL to extract the live cell fraction.

#### 3.6.6. Flow Cytometry (FC)

All samples were analyzed on a Navios flow cytofluorimeter (Beckman Coulter, Brea, CA, USA) equipped with three diode lasers (405, 488, and 638 nm). To distinguish single cells from aggregates and subsequently discriminate the aggregates from the analysis, the following combinations of direct (the value proportional to the cell’s size, forward scattering (FS)) and lateral (the value characterizing cell’s structure, side scattering (SS)) light scattering signals were used: the peak intensity compared with the integral signal’s intensity determined by FS or SS, and the flight time compared with the integral signal’s intensity determined by FS or SS. At least 30,000 single cells were analyzed in each sample. The results were analyzed using Kaluza software (Beckman Coulter, Brea, CA, USA).

**Figure 12:**
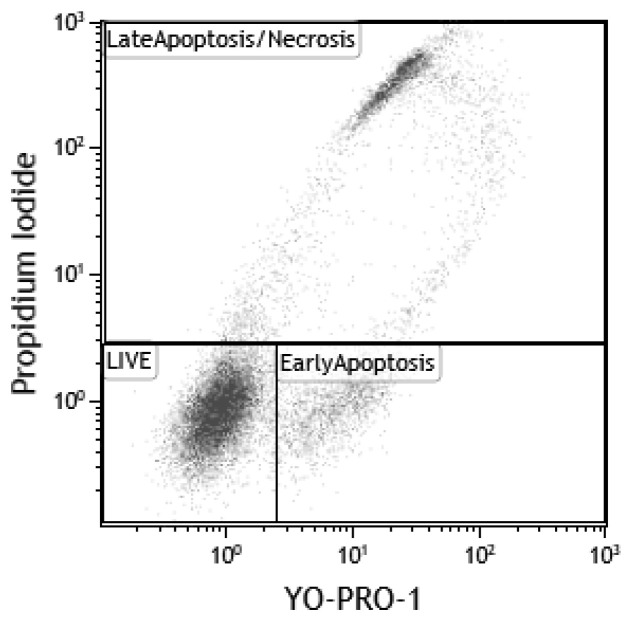
The gating tactics in the viability analysis of the TNR-1 cell line.

When assessing the degree of viability based on YO-PRO1 and PI fluorescence, the entire cell pool was divided into three populations: live cells (YO-PRO1−/PI−), cells in the early stages of apoptosis (YO-PRO1+/PI−) and cells in the late stages of apoptosis/necrosis (YO-PRO1+/PI+). The number of cells in these populations was expressed as a percentage of the total number of cells. The gating tactics are shown in Figure 12.

The average CD54-FITC fluorescence signal level (MFI, mean fluorescence intensity) was analyzed to assess the degree of cell activation. The results are presented in units of fluorescence. The analysis was performed only for live cells (DAPI-), as described previously [27].

To exclude the probability of aggregates of the studied nanoparticles entering the analysis zone, a calibration experiment was conducted in which nanoparticle solutions of different concentrations in the culture medium containing the studied substances were analyzed on a flow cytometer with similar settings to those used in the main experiment. It was found that the nanoparticles did not form aggregates that were registered by the device at the given settings and within the concentration limits used in the experiment.

Statistical processing was performed by using the MS Excel software package (Microsoft, USA) and GraphPad Prism 6.01 (GraphPad Software Inc., San Diego, CA, USA). The results are presented as the mean ± standard error of mean (mean ± SEM). Two-factor analysis of variance was used to compare quantitative values [28]. Tukey’s highly significant difference (HSD) test was used for further pairwise comparisons [29].

The results of a two-factor statistical analysis of the effects of NSMPs from the Nobel Replace and Alpha-Bio systems on activation, early and late apoptosis, and cell viability are presented in the Supplementary Materials (Figures S3–S10).

## 4. Conclusions

The present clinical case and the results of a histological analysis of soft tissue biopsy specimens were the basis for multidisciplinary studies. It was shown that with the presence of a functional load in the process of dental implants’ osseointegration, the formation of microparticles in the compositions of fibrous capsules took place. X-ray and electron microscopy established that the composition of the detected nano- and microparticles corresponded to the composition of the dental implants. The presence of nanoparticles in the supernatants obtained from the surfaces of the dental implants of the two manufacturers’ systems was confirmed by DLS.

As a result of the studies, the involvement of NSMP in both the physiological and pathophysiological processes of inflammation was confirmed. The presence of an autoimmune component in the pathogenesis of chronic immunopathological inflammation in tissues (mucositis and peri-implantitis) was experimentally proved.

By transferring the study to an in vitro model, it was possible to study the effect of nanoparticles of the “bioinert” TiO_2_ compound on human immune system cells in an experiment. Thus, a critical dose of nanoparticles leading to their coagulation to micron sizes under conditions of chronic inflammation was confirmed.

This suggestion requires further multidisciplinary research in terms of improving preventive, diagnostic and therapeutic algorithms for the management of patients during implant treatment.

## Figures and Tables

**Table 1:** Quantitative parameters of the microinclusions.

	Volume, µm^3^	*EqD*, µm	Linear Absorption Coefficient µ, mm^−1^
Min	729	11.2	0.22
Max	6,833,646	235.4	0.6
Mean	197,803	51.6	0.27

**Table 2:** Analysis of the expression levels of the CD54 molecule on the surface of THR-1 cells after 24 h of incubation with NSMP from the Alpha-Bio system.

	Control	Alpha-Bio 1/20	Alpha-Bio 1/10	Alpha-Bio 1
Control	1.37 ± 0.04	1.66 ± 0.08	1.85 ± 0.06	4.53 ± 0.30
LPS	9.80 ± 1.09	11.79 ± 0.81	13.06 ± 1.12	19.4 ± 0.42
Plasma	2.16 ± 0.57	1.87 ± 0.12	2.94 ± 0.94	14.1 ± 2.06
LPS + plasma	25.8 ± 0.04	26.8 ± 0.60	25.7 ± 0.54	22.8 ± 0.96

**Table 3:** Analysis of the expression level of CD54 molecule on the surface of THR-1 cells after 24 h of incubation with NSMP from the Nobel Replace system.

	Control	Nobel Replace 1/20	Nobel Replace 1/10	Nobel Replace 1
Control	1.10 ± 0.08	1.32 ± 0.054	1.38 ± 0.024	2.15 ± 0.030
LPS	11.09 ± 0.43	14.41 ± 0.76	12.05 ± 0.36	19.20 ± 1.19
Plasma	1.00 ± 0.09	1.24 ± 0.11	1.21 ± 0.04	4.70 ± 1.47
LPS + plasma	25.9 ± 0.64	26.8 ± 1.69	26.9 ± 0.49	26.3 ± 2.60

**Table 4:** Analysis of the viability of ТНР-1 cells after 24 h of incubation with NSMP from the Alpha-Bio system.

		Control	Alpha-Bio 1/20	Alpha-Bio 1/10	Alpha-Bio 1
Control	Live	87.45 ± 0.48	89.86 ± 0.26	89.41 ± 0.72	64.10 ± 1.07
	Early apoptosis	5.94 ± 0.73	4.56 ± 0.12	5.08 ± 0.49	10.94 ± 0.25
	Late apoptosis/necrosis	6.61 ± 1.00	5.58 ± 0.25	5.51 ± 0.49	24.96 ± 0.83
LPS	Live	84.66 ± 0.62	86.68 ± 0.88	86.61 ± 1.09	68.6 ± 1.26
	Early apoptosis	8.04 ± 0.34	7.18 ± 0.94	6.36 ± 0.70	8.05 ± 0.85
	Late apoptosis/necrosis	7.30 ± 0.59	6.13 ± 0.20	7.04 ± 0.82	23.4 ± 2.11
Plasma	Live	92.0 ± 0.24	93.3 ± 0.21	91.9 ± 0.67	67.4 ± 2.34
	Early apoptosis	4.39 ± 0.12	3.51 ± 0.39	4.23 ± 0.28	6.88 ± 0.60
	Late apoptosis/necrosis	3.60 ± 0.33	3.23 ± 0.21	3.84 ± 0.40	25.7 ± 1.75
LPS + plasma	Live	89.2 ± 0.43	89.1 ± 0.56	90.5 ± 0.24	70.5 ± 4.64
	Early apoptosis	5.70 ± 0.31	5.37 ± 0.29	4.62 ± 0.30	4.50 ± 0.12
	Late apoptosis/necrosis	5.09 ± 0.57	5.53 ± 0.30	4.84 ± 0.52	25.0 ± 4.67

**Table 5:** Analysis of the viability of ТНР-1 cells after 24 h of incubation with NSMP from the Nobel Replace system.

		Control	Nobel Replace 1/20	Nobel Replace 1/10	Nobel Replace 1
Control	Live	85.69 ± 1.93	87.64 ± 1.33	86.44 ± 0.37	63.70 ± 1.35
	Early apoptosis	6.15 ± 0.18	6.22 ± 1.31	6.24 ± 0.17	10.20 ± 0.87
	Late apoptosis/necrosis	8.16 ± 1.77	6.14 ± 0.94	7.31 ± 0.51	26.10 ± 2.21
LPS	Live	81.34 ± 1.83	84.28 ± 0.60	84.29 ± 0.57	71.4 ± 0.30
	Early apoptosis	9.64 ± 1.53	9.26 ± 0.32	8.35 ± 0.59	8.08 ± 0.41
	Late apoptosis/necrosis	9.02 ± 0.69	6.46 ± 0.31	7.36 ± 0.08	20.5 ± 0.17
Plasma	Live	92.20 ± 0.14	90.8 ± 0.06	91.6 ± 0.76	72.23 ± 0.99
	Early apoptosis	4.03 ± 0.23	4.72 ± 0.23	4.47 ± 0.64	5.86 ± 0.18
	Late apoptosis/necrosis	3.77 ± 0.35	4.50 ± 0.25	3.97 ± 0.21	21.9 ± 0.82
LPS + plasma	Live	87.8 ± 0.40	85.6 ± 0.74	86.4 ± 0.39	65.2 ± 5.85
	Early apoptosis	5.89 ± 0.89	6.39 ± 0.48	6.21 ± 0.39	3.27 ± 0.33
	Late apoptosis/necrosis	6.28 ± 0.72	8.04 ± 0.43	7.38 ± 0.19	31.5 ± 5.55

## Supplementary Materials

The supporting information can be downloaded at: https://www.mdpi.com/article/10.3390/ijms232415797/s1.
