# Supplementary material for: Immunopathological Inflammation in the Evolution of Mucositis and Peri-Implantitis
Source: Int J Mol Sci. 2022 Dec 13;23(24):15797. doi: 10.3390/ijms232415797 (PMC9779511; doi:10.3390/ijms232415797)
Supplement: Supplementary file 1 [file ijms-23-15797-s001.zip › ijms-2015124-supplementary.pdf]

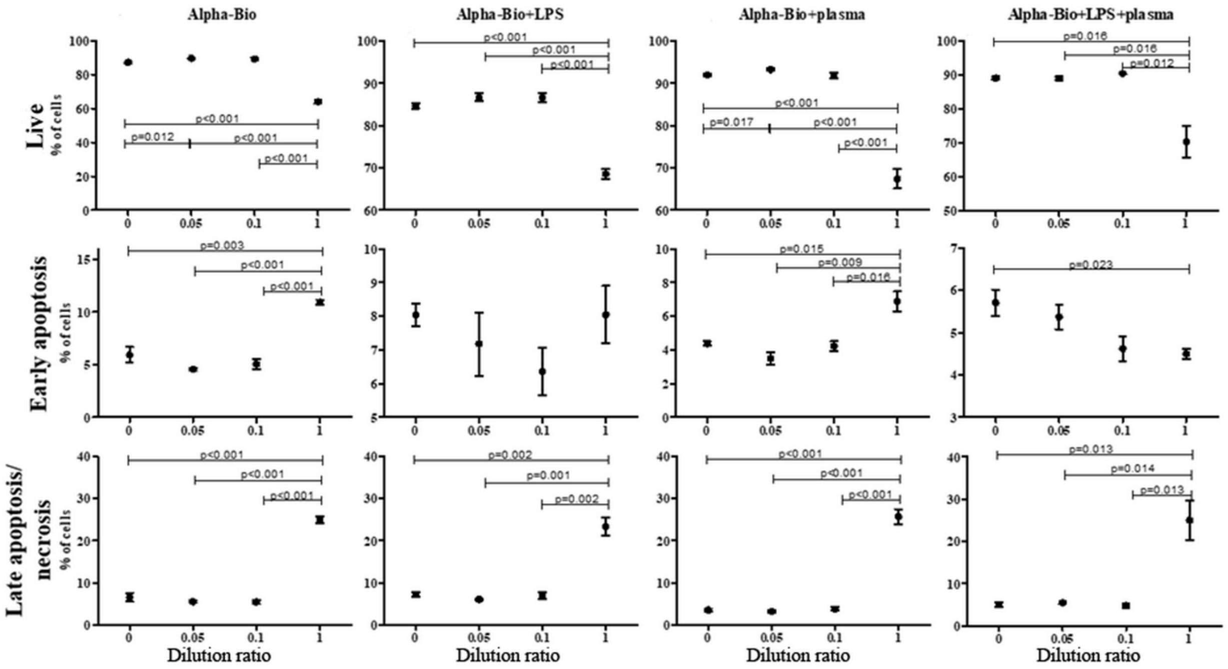

Supplementary Figure S1. Viability of THP-1 cells after 24 h of incubation with NSMP from the Alpha-Bio system. LPS, incubation with NSMP and PBS as a co-stimulator; Plasma, incubation with NSMP and blood plasma from healthy donors as a co-stimulator; LPS+Plasma, incubation with NSMP and a combination of LPS and blood plasma from healthy donors as co-stimulators.

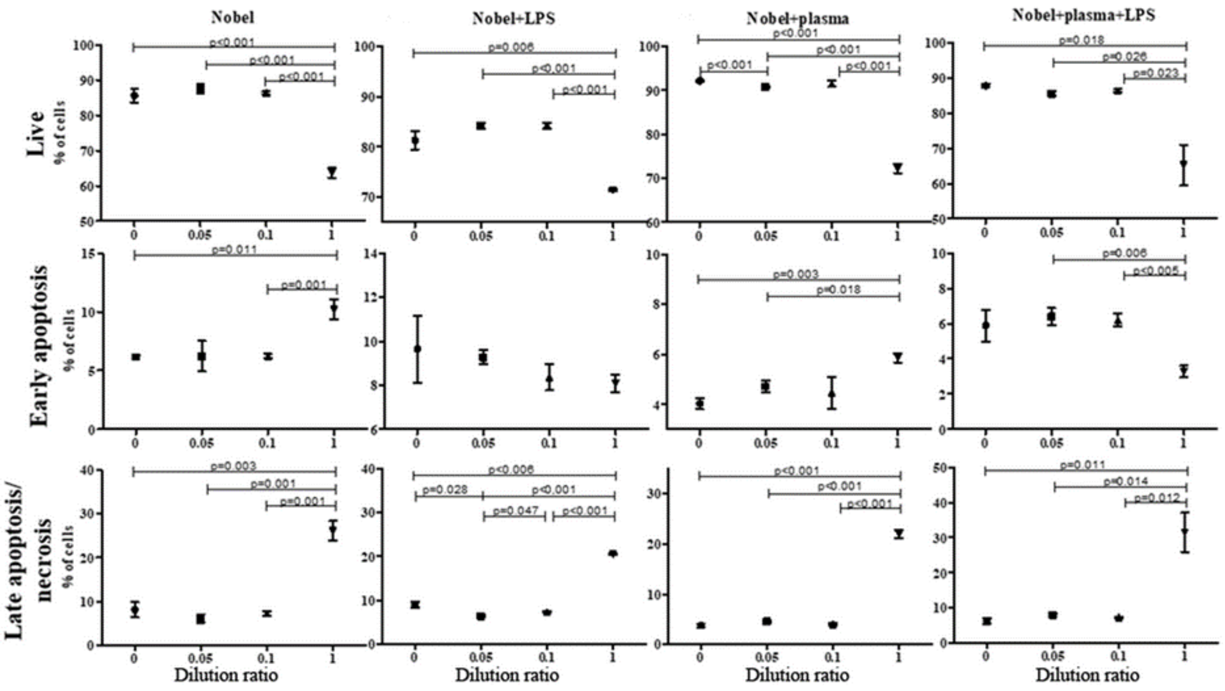

Supplementary Figure S2. Viability of THP-1 cells after 24 h of incubation with NSMP from the Alpha-Bio system. LPS, incubation with NSMP and PBS as a co-stimulator; Plasma, incubation with NSMP and blood plasma from healthy donors as a co-stimulator; LPS+Plasma, incubation with NSMP and a combination of LPS and blood plasma from healthy donors as co-stimulators.

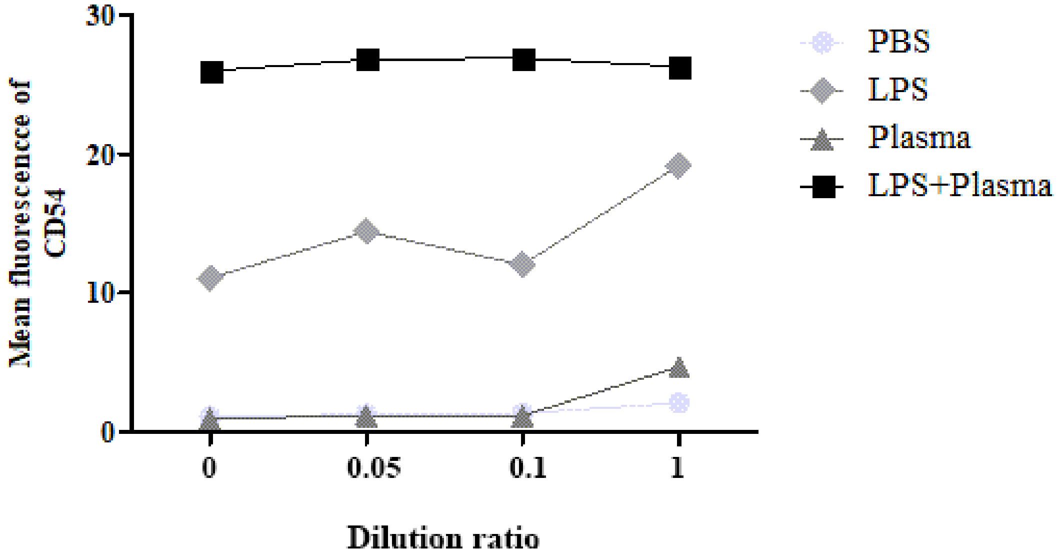

Supplementary Figure S3. Results of a two-factor statistical analysis of the effect of Nobel Replace NSMP on THP-1 activation.

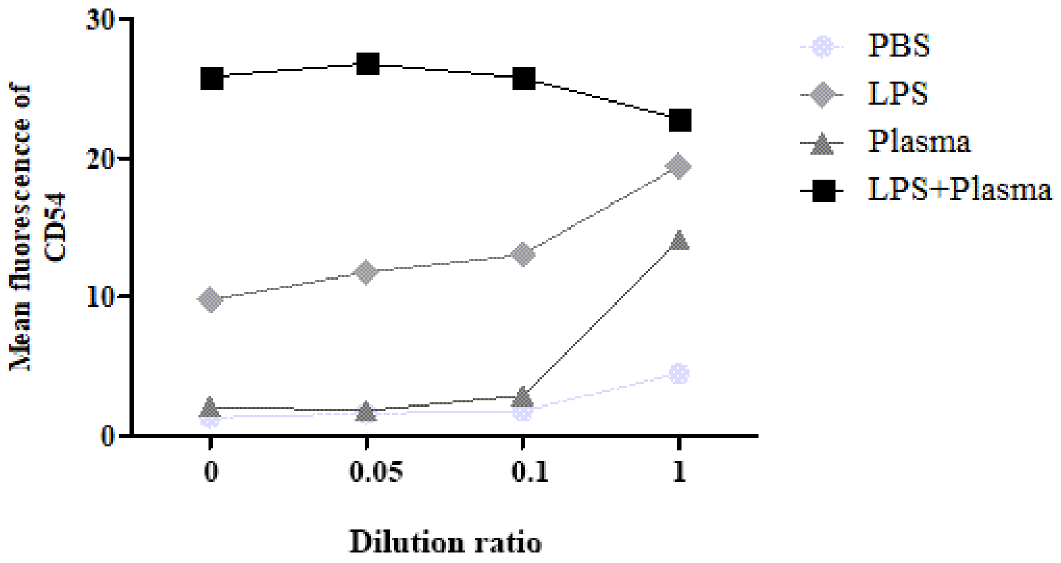

Supplementary Figure S4. Results of a two-factor statistical analysis of the effect of Alpha-Bio NSMP on THP-1 activation.

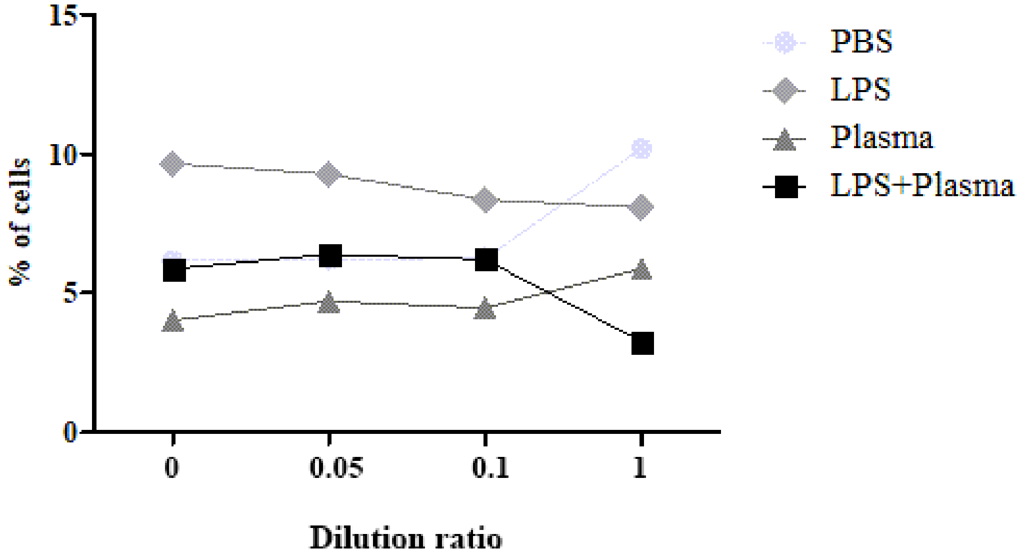

Supplementary Figure S5. Results of a two-factor statistical analysis of the effect of Nobel Replace NSMP on THP-1 early apoptosis.

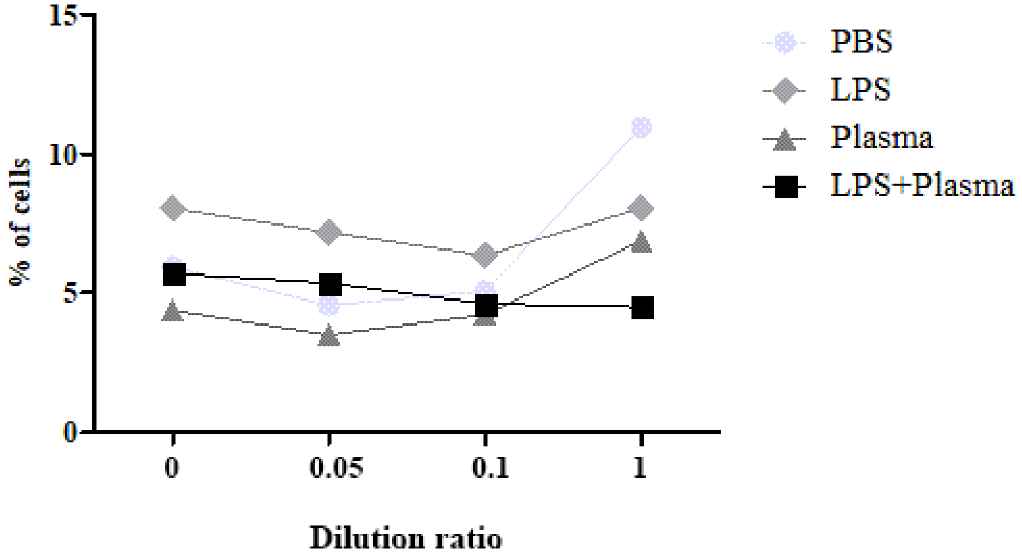

Supplementary Figure S6. Results of a two-factor statistical analysis of the effect of Alpha-Bio NSMP on THP-1 early apoptosis.

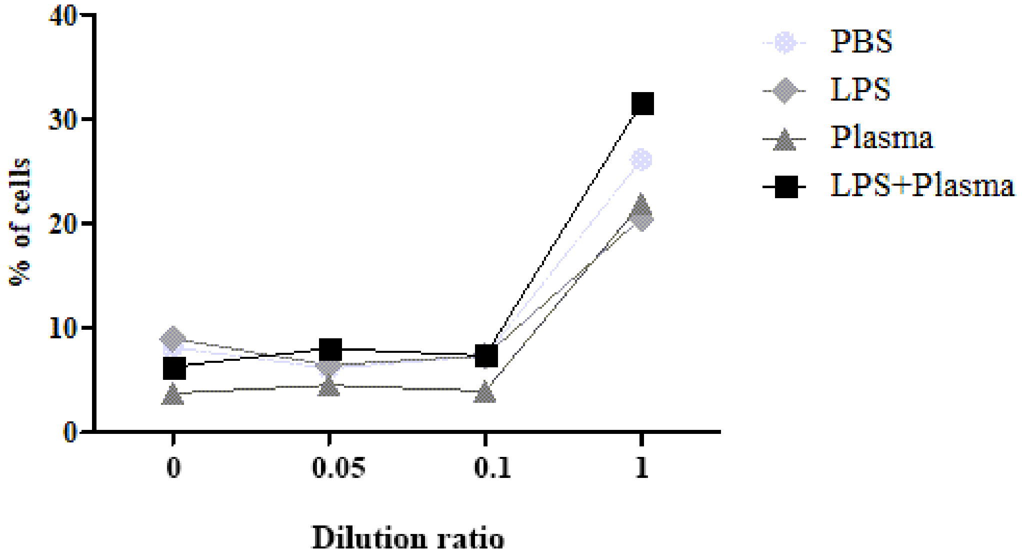

Supplementary Figure S7. Results of a two-factor statistical analysis of the effect of Nobel Replace NSMP on THP-1 late apoptosis.

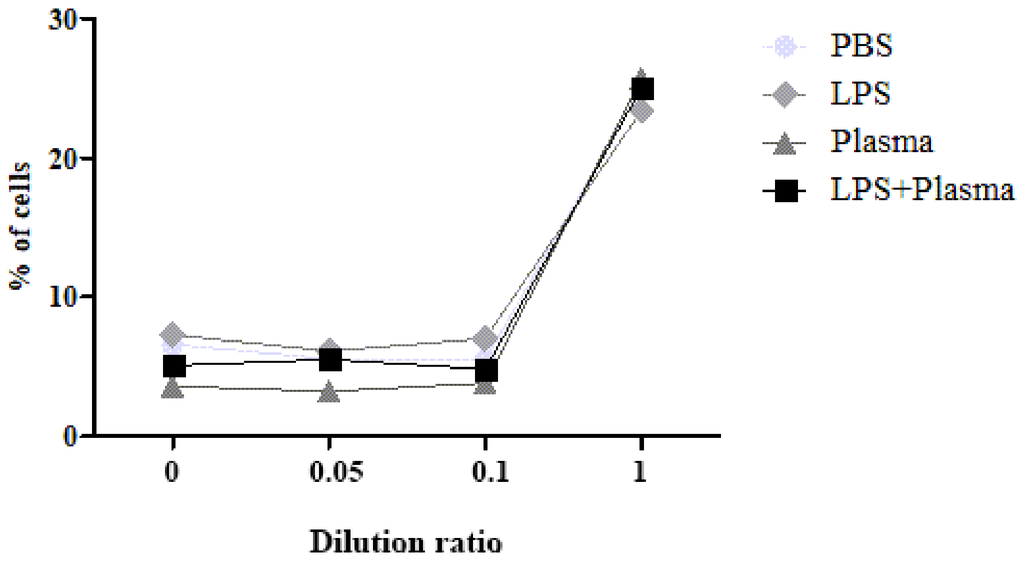

Supplementary Figure S8. Results of a two-factor statistical analysis of the effect of Alpha-Bio NSMP on THP-1 late apoptosis.

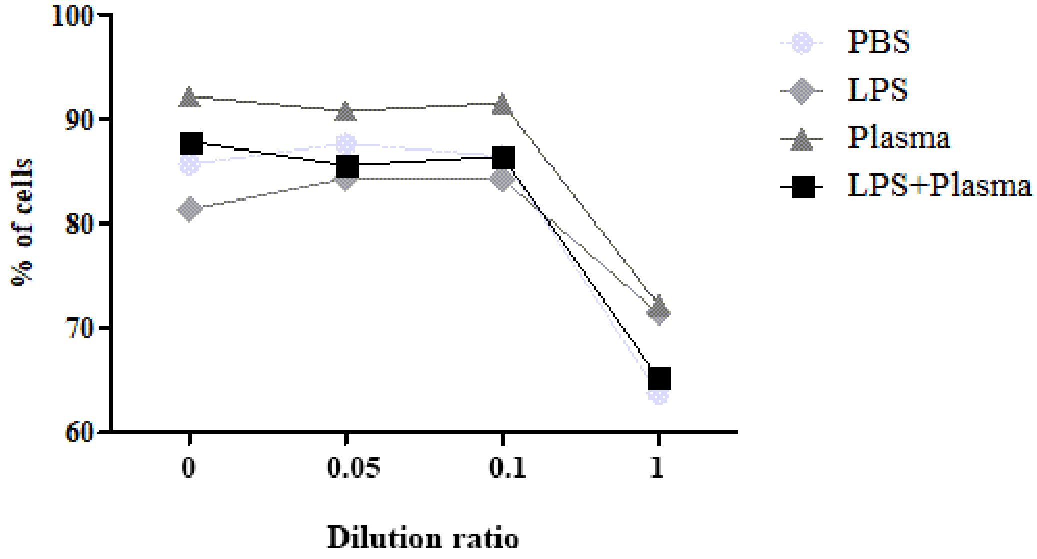

Supplementary Figure S9. Results of a two-factor statistical analysis of the effect of Nobel Replace NSMP on THP-1 viability.

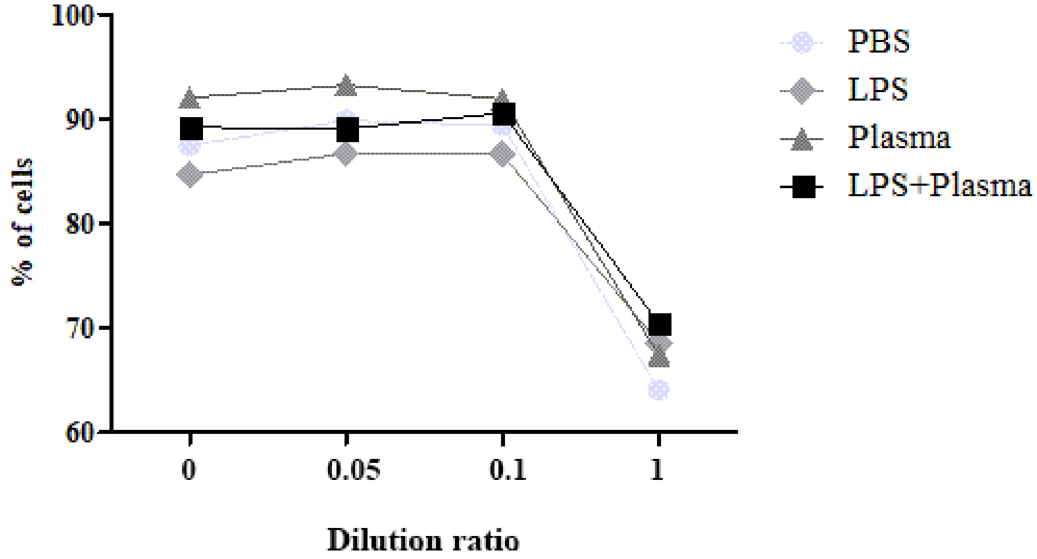

Supplementary Figure S10. Results of a two-factor statistical analysis of the effect of Alpha-Bio NSMP on THP-1 viability.
